# Decision-to-delivery interval of emergency cesarean section in Uganda: a retrospective cohort study

**DOI:** 10.1186/s12884-020-03010-x

**Published:** 2020-05-27

**Authors:** Noemi J. Hughes, Imelda Namagembe, Annettee Nakimuli, Musa Sekikubo, Ashley Moffett, Charlotte J. Patient, Catherine E. Aiken

**Affiliations:** 1 School of Clinical Medicine, University of Cambridge, NIHR Cambridge Comprehensive Biomedical Research Centre, Cambridge, CB2 0SW UK; 2 Department of Obstetrics and Gynecology, Makerere University and Mulago National Referral Hospital, Kampala, Uganda; 3 Department of Pathology and Centre for Trophoblast Research, University of Cambridge, Cambridge, CB2 3EG UK; 4 Department of Obstetrics and Gynecology, Box 223, The Rosie Hospital, Cambridge, CB2 0SW UK; 5 University Department of Obstetrics and Gynecology, University of Cambridge, NIHR Cambridge Comprehensive Biomedical Research Centre, Cambridge, CB2 0SW UK

**Keywords:** Africa, Cesarean, Decision, Emergency, Obstetrics, Perinatal, Uganda

## Abstract

**Background:**

In many low and medium human development index countries, the rate of maternal and neonatal morbidity and mortality is high. One factor which may influence this is the decision-to-delivery interval of emergency cesarean section. We aimed to investigate the maternal risk factors, indications and decision-to-delivery interval of emergency cesarean section in a large, under-resourced obstetric setting in Uganda.

**Methods:**

Records of 344 singleton pregnancies delivered at ≥24 weeks throughout June 2017 at Mulago National Referral Hospital were analysed using Cox proportional hazards models and multivariate logistic regression models.

**Results:**

An emergency cesarean section was performed every 104 min and the median decision-to-delivery interval was 5.5 h. Longer interval was associated with preeclampsia and premature rupture of membranes/oligohydramnios. Fetal distress was associated with a shorter interval (*p* < 0.001). There was no association between decision-to-delivery interval and adverse perinatal outcomes (*p* > 0.05). Mothers waited on average 6 h longer for deliveries between 00:00–08:00 compared to those between 12:00–20:00 (*p* < 0.01). The risk of perinatal death was higher in neonates where the decision to deliver was made between 20:00–02:00 compared to 08:00–12:00 (*p* < 0.01).

**Conclusion:**

In this setting, the average decision-to-delivery interval is longer than targets adopted in high development index countries. Decision-to-delivery interval varies diurnally, with decisions and deliveries made at night carrying a higher risk of adverse perinatal outcomes. This suggests a need for targeting the improvement of service provision overnight.

## Summary box

A. ***What is already known?***

- In low human development index settings, emergency cesarean section is often associated with high rates of maternal and neonatal morbidity and mortality.
- Decision-to-delivery interval of emergency cesarean section is a modifiable factor which can influence perinatal outcomes.

B. ***What are the new findings?***

- Average decision-to-delivery intervals are long in this setting compared to international guidelines.
- There is no direct association between decision-to-delivery interval and risk of adverse outcomes.
- There is diurnal variation in both decision-to-delivery intervals and adverse outcomes, with both increasing overnight.

C. ***What do the new findings imply?***

- Reducing the diurnal variation in decision-to-delivery interval could improve perinatal outcomes of emergency caesarean section.
- We suggest this could be achieved by altering patterns of service provision to better support deliveries and decision-making overnight.

## Background

In many low and medium human development index (LM-HDI [1]) countries, the rate of maternal and neonatal morbidity and mortality is high [2, 3]. Preventing adverse perinatal outcomes is often critically time-dependent, however, demand can exceed capacity for prompt intervention in these settings wall]. Despite global initiatives for improvement, there remains considerable complexity in increasing obstetrics resource availability in LM-HDI settings [4, 5]. We considered whether improving patterns of service provision might provide an alternative strategy to reduce adverse perinatal outcomes in LM-HDI settings. Studies have shown that risk of adverse perinatal outcomes is associated with service provision factors such as obstetric staff working patterns [6, 7]. This has only, however, been studied extensively in well-resourced countries and so there is a need to investigate the modifiable patterns of service provision in low and medium human development index obstetric settings.

One aspect of service provision with the potential to influence perinatal outcome is the decision-to-delivery interval of emergency cesarean section [8]. National guidelines in the USA and UK suggest a target of 30 min after the decision to deliver by emergency cesarean section is established [9, 10]. Such guidelines, however, are not well-evidenced [11] and may not be feasible even in well-resourced obstetric settings [12]. Furthermore, whilst globally it is generally accepted that the decision-to-delivery interval should be kept to the minimum time achievable [13], there are currently no context-appropriate targets intended to minimise adverse outcomes in low and medium human development index countries.

Mulago National Referral Hospital is a government-run facility with over 2700 beds [14] in Kampala, Uganda. The birth rate has been reported to exceed 39,000 per annum [15]. Approximately 22% of all deliveries in the study centre are by caesarean section, of which ~ 85% are by emergency cesarean section [16]. This high volume combined with the socio-economic challenges [15], poor baseline health status [17, 18], and lack of antenatal care [19] experienced by mothers presenting to the hospital, has resulted in high rates of maternal and neonatal morbidity and mortality [20, 21]. Studies from this setting have shown that obstetric service provision is not uniform during a 24-h shift cycle [22]. We therefore aimed to investigate whether the modifiable factor of decision-to-delivery interval is associated with adverse perinatal outcomes.

## Methods

Mothers who delivered a singleton pregnancy, at viable gestational age (≥24 completed weeks), by emergency cesarean section during the period of June 2017 were included in the study. We analysed their full medical records for the entire delivery episode from admission to discharge. Data was collected contemporaneously in fully anonymised form and the data used for analysis is summarised in Table 1. Mothers who died following emergency cesarean section were excluded from the study (*n* ≤ 3), due to possession of their records by other investigatory authorities. Cases of intrauterine death in which the fetus was thought to have demised prior to presentation at the hospital were also excluded, as this outcome could not have been affected by decision-to-delivery interval. The diagnosis of fetal demise prior to presentation was made according to the contemporaneous judgement of the attending clinician. The diagnosis was based primarily on the macerated appearance of the fetus after delivery, but also took into account factors such as whether the fetal heart was ever auscultated and the maternal history. A typical shift at the study centre is staffed by an attending obstetrician, 3–4 resident obstetricians, and one intern. For all included deliveries, the decision was made by an attending doctor to deliver on an emergency basis by caesarean section. Neonatalogy services are available at the study centre.

**Table 1:** Summary of data extracted from contemporaneous medical notes

Data	Details
Maternal age	Self-reported by mother or referring clinician.
Gestational age	Calculated from the date of last menstrual period stated by mother or symphysial-fundal height. Routine first trimester US is not available in this context.
Previous cesarean section	Evidenced by an existing abdominal scar, with maternal report.
Comorbidities (composite factor)	One or more of HIV, active malaria and sickle-cell crisis as stated by mother or diagnosed by attending clinician.
Pre-eclampsia	Diagnosed according to modified ACOG guidelines [16] – blood testing is not routinely available for investigating suspected pre-eclampsia, therefore the criteria based on biochemical results were not applied.
Antepartum haemorrhage	Any fresh vaginal blood loss reported by the mother prior to delivery
Premature rupture of membranes / oligohydramnios	Premature rupture of membranes based on maternal history, oligohydramnios was diagnosed by clinicians on the basis of clinical examination +/− ultrasound scan
Uterine rupture	Based on clinical suspicion at the time of decision-making
Obstructed labour	Diagnosed by the decision-making clinician based on examination (e.g. excessive fetal caput, haematuria) or history (e.g. length of time in labor)
Fetal distress	Diagnosed by the decision-making clinician based on clinical suspicion e.g. meconium stained liquor or decelerations on intermittent auscultation. Continuous fetal monitoring, and fetal blood sampling were not available
Malpresentation	Diagnosed by the delivering clinician
Cord prolapse	Diagnosed by the delivering clinician
Decision	Date and time at which the decision to deliver by emergency cesarean section was recorded in the contemporaneous medical notes.
Delivery	Date and time at which the neonate was delivered according to the operation note
Decision-to-delivery interval	Calculated to the nearest minute
Adverse maternal outcome (composite)	One or more: confirmed uterine rupture at delivery, severe postpartum haemorrhage (≥1 L blood), emergency hysterectomy, admission to the High-Dependency Unit or obstetric palsy
Neonatal APGAR scores	Recorded at 1 and 5 min
Stillbirth	Viable baby born with no signs of life that was believed to have been alive at admission to hospital
Neonatal death	Live birth at viable gestational age, followed by death prior to hospital discharge
Perinatal death (composite)	All stillbirths and neonatal deaths (defined as above)
Adverse neonatal outcome (composite)	One or more of birth asphyxia, resuscitation, birth trauma and respiratory distress
Gravidity	Self-reported number of previous pregnancies
Parity	Self-reported number of previous deliveries ≥24 weeks
Birth weight	Recorded to the nearest 100 g
Neonatal sex	As recorded in contemporaneous medical record

Using binary logistic regression, we compared the characteristics of deliveries occurring during the day to those during the night and those during weekends to weekdays. Using univariate and multivariate Cox proportional hazard models, we assessed the relationship between both maternal risk factors and indications for each emergency cesarean section and the decision-to-delivery interval. These models accounted for time-at-risk of adverse outcomes. Using binomial regression models, we assessed the relationship between adverse perinatal outcomes and the decision-to-delivery interval. Using generalised additive models (in which all events were considered equivalent), we assessed the relationship between (i) time of decision, (ii) time of delivery, and (iii) decision-to-delivery interval, and adverse outcomes. The generalised additive models incorporate a nonlinear term for event time on the risk of each adverse outcome and this was estimated using cubic splines. All events included in the modelling were considered equivalent. Using non-parameter models avoids the requirement to make any assumptions about the nature of the relationship between the timing of an event and the risk of an adverse outcome. The risk of an event at any particular time, relative to the average population risk, can therefore be assessed from the graphical representations of the models presented in the figures.

All multivariate models were adjusted for covariates selected on the basis of clinical relevance and using Akaike Information Criterion (AIC) to optimise model fit. Statistical significance of the nonlinear effect of time of delivery was assessed using a likelihood-ratio test. To summarise our findings, Kaplan-Meier curves were constructed to represent the decision-to-delivery interval of the entire population and of relevant sub-cohorts. Our findings were considered statistically significant at an alpha level of 0.05. Power calculations were performed by Monte Carlo simulation. All analyses were conducted using the R statistical software package version 3.5.1.

## Results

**Fig. 1:**
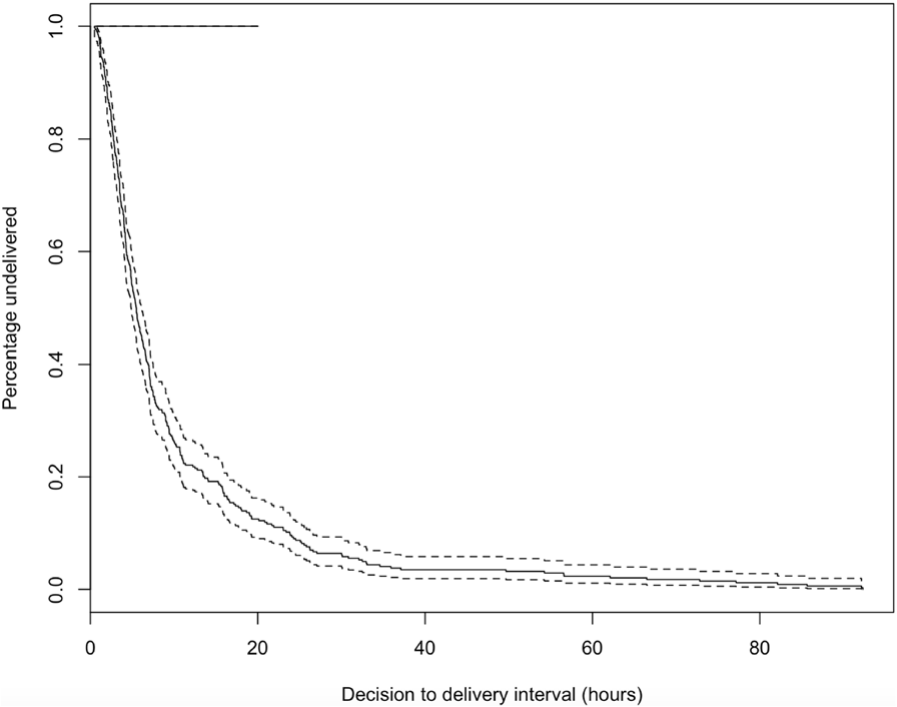
Percentage of emergency cesarean section completed by time from decision-making. Median decision-to-delivery interval: 5.5 h (IQR 3.3–10.7 h). Mean decision-to-delivery interval: 10.2 h (S.D. ± 13.9 h)

During the study period of June 2017, 412 mothers underwent an emergency cesarean section at Mulago Hospital. Of these, 396 delivered a singleton pregnancy at viable gestational age (≥24 completed weeks) and 349 of these records had complete information regarding the decision-to-delivery interval. 5 observations were removed from the time-dependent analysis on the basis that their status as an emergency was reversed prior to delivery. In these 5 cases, the neonate was delivered more than 4 days after the initial decision time. A delivery by emergency cesarean section occurred on average every 104 min (median 13.7 per day) throughout the entire study period. The median decision-to-delivery interval was 5.5 h, with interquartile range 3.3–10.7 and range 0.5–92.3 h (Fig. 1). 2% (7/344) of neonates were delivered within 1 h of decision-making.

**Fig. 2:**
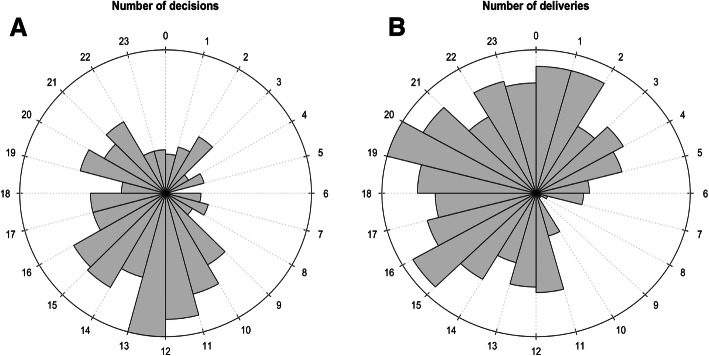
Distribution of emergency cesarean section throughout the 24-h period. **a**) Number of decisions for emergency cesarean section by hour. There was significant variation in the average number of decisions per hour throughout the day (*p* < 0.001). **b**) Number of deliveries by emergency cesarean section. There was significant variation in the average number of deliveries per hour throughout the day (*p* < 0.001)

**Fig. 3:**
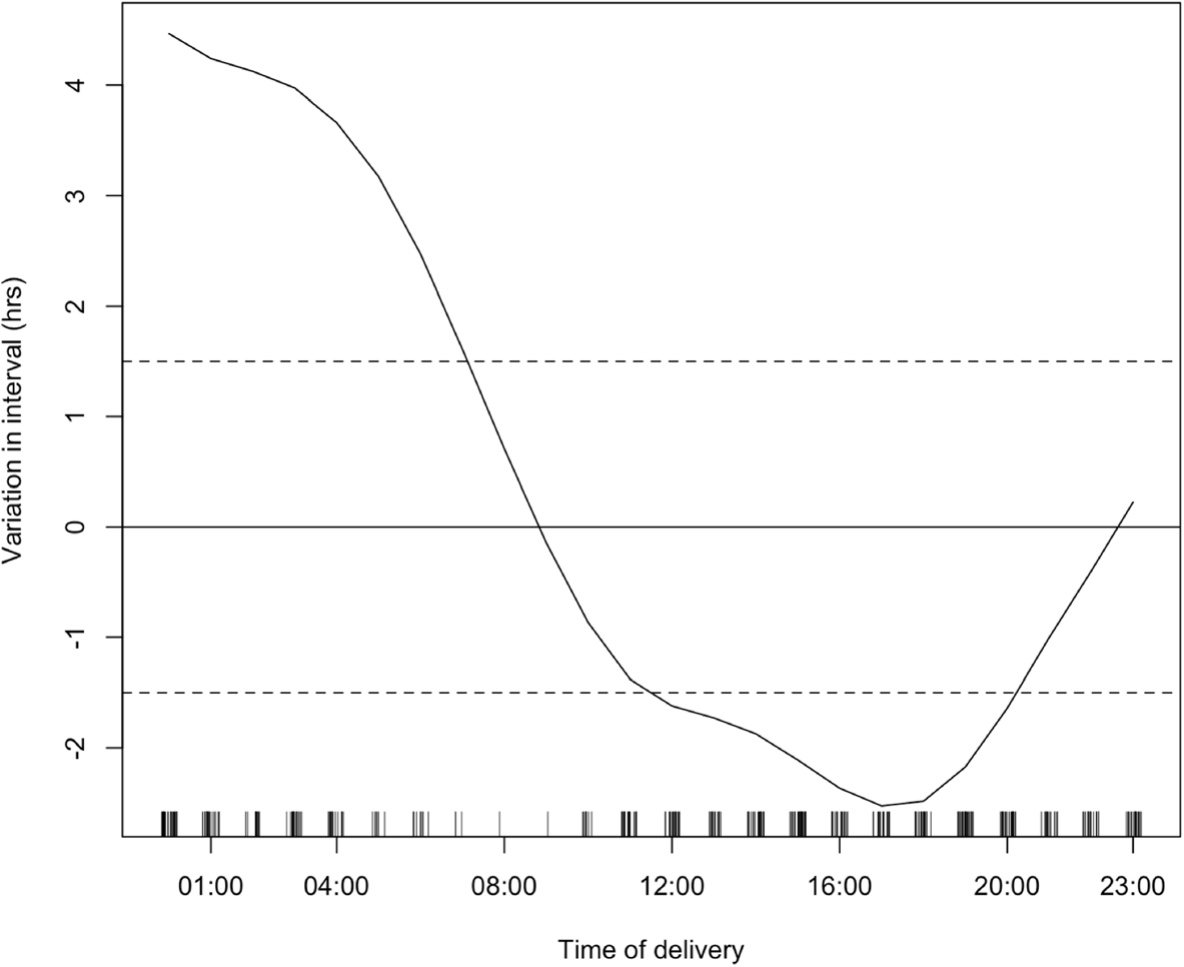
Average decision-to-delivery interval by time of delivery. Solid line: median decision-to-delivery interval. Dashed lines: ± standard errors, decision-to-delivery interval varies significantly over the 24-h period; *p* < 0.01

The average number of decisions made per hour varied significantly throughout the day (minimum: 0.03 decisions per hour 05:00–06:00, maximum: 1.1 decisions per hour between 12:00–13:00, *p* < 0.001, Fig. 2a). The average number of deliveries also varied significantly (minimum 0.03 deliveries per hour 08:00–10:00, maximum 0.87 deliveries per hour between 19:00–20:00, *p* < 0.001, Fig. 2b). The average length of decision-to-delivery interval also varied significantly throughout the day (minimum 3.3 h 16:00–17:00, maximum 9.4 h 01:00–02:00, *p* < 0.01, Fig. 3). Specifically, mothers who delivered during the night (between 0:00 and 08:00) waited on average 2 h longer for their emergency cesarean section compared to the rest of the cohort. There was a 5.6-h difference in average length of interval between those delivering during the longest wait times (00:00 to 08.00) and shortest wait times (12,00 to 20.00) (Fig. 3). There was no significant difference in the interval on any particular day of the week or at the weekend.

None of the risk factors known prior to birth, including preeclampsia, were significantly associated with shorter decision-to-delivery intervals using univariate analysis (Table 2). Longer interval was associated only with preeclampsia (*p* < 0.05) and premature rupture of the membranes / oligohydramnios (*p* < 0.01) on univariate analysis. After adjustment for relevant covariates, both preeclampsia (HR 0.61; CI 0.38–0.97, *p* < 0.05) and premature rupture of the membranes / oligohydramnios (HR 0.60; CI 0.37–0.96, p < 0.05) retained significance.

**Table 2:** Factors known prior to delivery

Maternal factor		Characteristic (*n* = 344)	Impact on decision-to-delivery interval	Significance
Age		25.4 ± 5.1	0.99 (0.97–1.02)	0.59
Gestational age		37.57 ± 2.0	1.05 (0.99–1.11)	0.06
Parity	0	130 (37.8%)	Ref	
	1	81 (23.5%)	0.93 (0.71–1.23)	0.63
	2	65 (18.9%)	0.97 (0.71–1.31)	0.83
	≥3	68 (19.8%)	1.02 (0.75–1.34)	0.91
Co-morbidities	No	339 (98.5%)	Ref	
	Yes	4 (1.5%)	1.25 (0.52–3.03)	0.62
Previous cesarean section	No	196 (57%)	Ref	
	Yes	148 (43%)	0.92 (0.74–1.14)	0.44
Previous poor neonatal outcome	No	334 (97.1%)	Ref	
	Yes	10 (2.9%)	1.17 (0.61–2.21)	0.64
Preeclampsia	No	323 (93.9%)	Ref	
	Yes	21 (6.1%)	0.62 (0.39–0.98)	0.04*
APH	No	325 (94.5%)	Ref	
	Yes	19 (5.5%)	1.29 (0.81–2.05)	0.28
PROM/ oligohydramnios	No	322 (93.6%)	Ref	
	Yes	22 (6.4%)	0.55 (0.36–0.86)	< 0.01**

Numeric characteristics are shown as mean ± standard deviation. Categorical characteristics are shown as n (%). Impact on decision-to-delivery interval is represented by the hazard ratio and confidence intervals from a Cox proportional hazards model conditioned only on the characteristic of interest. Significance is the *p* value derived from the same model. *p < 0.05, **p < 0.01

**Fig. 4:**
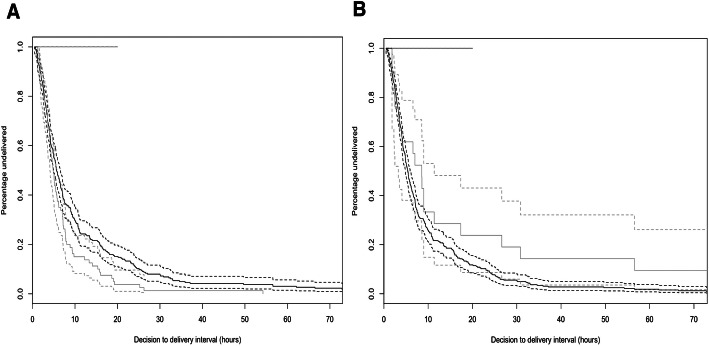
decision-to-delivery interval by indication for emergency cesarean section. **a**) Solid grey line: deliveries where fetal distress was an indication, dashed grey lines: 95% confidence intervals, solid black line: all deliveries without fetal distress as an indication, dashed black lines: 95% confidence intervals. *p* < 0.05. **b**) Solid grey line: deliveries where preeclampsia was an indication for emergency cesarean section, dashed grey lines: 95% confidence intervals, solid black line: all deliveries without preeclampsia as an indication, dashed black lines: 95% confidence intervals. *p* < 0.05

We examined whether individual indications for emergency cesarean section were associated with the decision-to-delivery interval (Table 3) with univariate analysis. Fetal distress was associated with a shorter interval (*p* < 0.05, Fig. 4a) whilst preeclampsia was associated with a longer interval (p < 0.05, Fig. 4b). After adjustment for relevant covariates in multivariate models, fetal distress (HR 1.63, CI 1.23–2.15; *p* < 0.001), previous cesarean section (HR 1.66, CI 1.24–2.21; *p* < 0.01), malpresentation (HR 1.78, CI 1.17–2.69; p < 0.01), antepartum haemorrhage (HR 1.56, CI 1.00–2.43, *P* < 0.05) and impending uterine rupture (HR 1.85, CI 1.24–2.78; *p* < 0.05) were all significantly associated with shorter interval.

**Table 3:** Indications for emergency cesarean section

Indication for emergency cesarean section	Number (%, *n* = 344)	Average decision-to-delivery interval	Significance
Previous cesarean section (no suspicion of rupture)	87 (25.3%)	5.1 (3.0–9.4)	0.08
Previous cesarean section (suspicion of rupture)	35 (10.2%)	5.2 (3.3–8.3)	0.19
Obstructed labour	172 (50.0%)	5.5 (3.5–10.7)	0.70
Fetal distress	80 (23.3%)	4.9 (3.3–7.7)	< 0.05*
Malpresentation (54% breech)	26 (7.6%)	4.0 (2.7–7.0)	0.06
Antepartum haemorrhage / placenta praevia / accreta	23 (6.7%)	3.3 (1.9–11.0)	0.34
Preclampsia	21 (6.1%)	8.6 (3.2–17.4)	< 0.05*
Cord prolapse	5 (1.5%)	6.3 (4.9–6.6)	0.65
Other	10 (2.9%)	7.3 (6.0–27.8)	0.07

The number of mothers with each indication for emergency cesarean section along with the percentage of the analytic cohort is shown. More than one indication was present in many cases. The median decision-to-delivery interval and IQR range are shown for each indication. The impact of each indication on decision-to-delivery interval is represented by the *p*-values from a Cox proportional hazards model conditioned only on the indication of interest. Significance is the p value derived from the same model. *p < 0.05

**Fig. 5:**
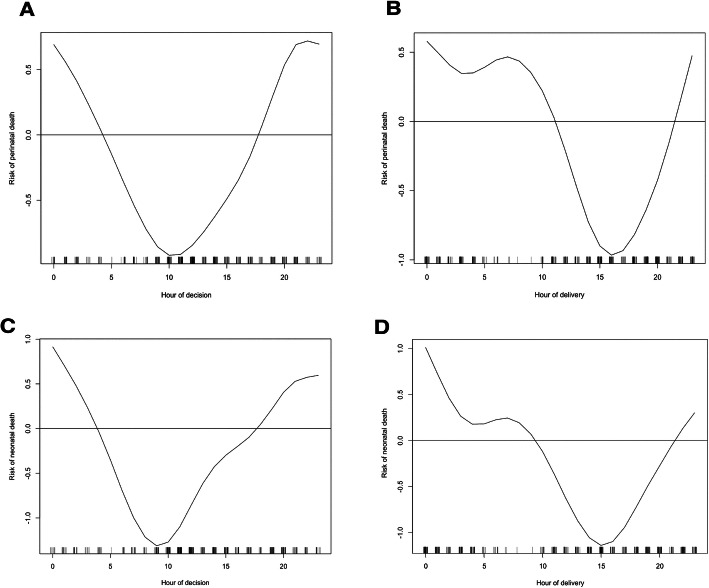
Risk of adverse perinatal outcomes by time of day. **a** Risk of perinatal death by hour of decision making. The risk of perinatal death was significantly higher than average for neonates where the decision to deliver by emergency cesarean section was made at night (20:00–02:00) and significantly lower than average where the decision was made in the morning (08:00–12:00), *p* < 0.01. **b** Risk of perinatal death by hour of delivery. The risk of perinatal death was significantly higher than average for neonates delivered at night (24:00–08:00) and significantly lower than average where delivery was in the afternoon (14:00–18:00), *p* < 0.05. **c**) Risk of neonatal death by hour of decision making. The risk of neonatal death was significantly higher than average for neonates where the decision to deliver by emergency cesarean section was made at night (24:00–02:00) and significantly lower than average where the decision was made in the morning (06:00–12:00), *p* < 0.05. **d**) Risk of neonatal death by hour of delivery. The risk of neonatal death was not significantly higher than average for neonates delivered at night, however, was significantly lower than average where delivery was in the afternoon (13:00–17:00), *p* < 0.05

None of the maternal or fetal adverse outcomes measured showed a linear relationship with the decision-to-delivery interval (Table 4). There was, however, a significant association between the timing of a decision or delivery and adverse perinatal outcomes. This included the outcomes of both perinatal (Fig. 5a-b**;*p*** **< 0.05**) and neonatal (Fig. 5c-d**;*p*** **< 0.05**) death. The risk of both perinatal death and neonatal death varied by > 50% between the highest and lowest risk periods (Fig. 5a-d). The lowest risk decision period was in the morning between 08:00 and noon (*p* < 0.01), whereas the lowest risk delivery period was in the afternoon between noon and 20:00 (*p* < 0.05). The highest risk decisions and deliveries both occurred between 22:00 and 04:00. The risk of both perinatal death (*p* < 0.05) and neonatal death (p < 0.05) showed significantly stronger association with the decision time than delivery time.

**Table 4:** Outcomes of delivery

Delivery outcome	Number (%)	Influence of decision-to-delivery interval	Influence of decision time	Influence of delivery time
Adverse maternal outcome	16 (4.7%)	*p* = 0.26	*p* = 0.61	*p* = 0.54
Fresh stillbirth	13 (3.8%)	*p* = 0.15	*p* = 0.16	*p* = 0.07
Neonatal death	21 (6.4%)	*p* = 0.85	*p* < 0.05*	*p* = 0.07
Perinatal death	35 (10.2%)	*p* = 0.68	*p* < 0.01**	*p* < 0.05*
Admission to the Special Care Baby Unit	78 (23.6%)	*p* = 0.75	*p* = 0.59	*p* = 0.53
APGAR < 7 at 1 min	76 (23.0%)	*p* = 0.57	*p* = 0.16	*p* = 0.47
APGAR < 7 at 5 mins	77 (23.3%)	*p* = 0.56	*p* = 0.13	*p* = 0.39
Other adverse neonatal outcome	47 (14.2%)	*p* = 0.24	*p* < 0.05*	*p* < 0.05*

The number of mothers who experienced each adverse outcome is shown along with the percentage. For outcomes that can apply to all deliveries (perinatal death, stillbirth, adverse maternal outcomes) the total was *n* = 344. For outcomes that apply only to live born infants, the total was *n* = 330. More than one adverse outcome was present in some cases. The influence of decision-to-delivery interval is represented by p-values derived from logistic regression models, in which the risk of outcome is conditioned upon the length of the interval in hours. The influence of decision time and delivery time are the *p*-values derived from generalised additive models with a non-parametric effect for the time of day at decision and delivery respectively. All models were adjusted for the sex and gestational age-specific centile of the neonate’s birth weight. *p < 0.05, **p < 0.01

## Discussion

In this obstetric setting, the average decision-to-delivery interval was 5.5 h, with only 2% of babies delivered within an hour of decision-making. Women with certain indications were delivered more quickly once the decision for emergency cesarean section was made. Fetal distress, malpresentation, antepartum haemorrhage, and previous cesarean section (both with and without concern regarding impending uterine rupture) were prioritised over other indications. Whilst there was no association between the length of the decision-to-delivery interval and adverse perinatal outcomes, both the interval and risk of perinatal mortality showed significant diurnal variation. The time of decision-making was better correlated with the risk of adverse perinatal outcome that the time of delivery.

Accounting for the time taken to clean and restock the theatres between cases, we observed a remarkable continuous rate of emergency surgery in this LM-HDI setting. The average decision-to-delivery interval was significantly longer than targets adopted in well-resourced obstetric settings [10] as well as the average interval reported in other low resource contexts globally [13, 23]. However inconsistent categorisation of cases as ‘emergencies’ [24] and different obstetric populations complicate international comparisons.

There was significant diurnal variation in decision-to-delivery interval and risk of perinatal death, which may reflect the fluctuating availability of senior clinicians during a 24-h period. As in many obstetric settings globally, the most experienced obstetricians at Mulago Hospital are available for ward rounds and decision-making during the day, however, not overnight. In line with this, the times of shortest interval occurred during normal working hours (12:00–20:00). The period with fewest decisions for emergency cesarean section were made (22:00–02:00) corresponded to the times of highest perinatal mortality, whilst the period when the rate of decision-making was increasing most rapidly (09:00–13:00) was associated with the lowest risk.

Reducing the average decision-to-delivery interval by 5 h in keeping with the 30-min targets set elsewhere [9, 10] is unlikely to be feasible in our already under-resourced study setting. Moreover, existing guidelines are not well-evidenced [11] with little direct evidence of benefit even in well-resourced obstetric setting. We did not find evidence of a direct relationship between longer interval and adverse perinatal outcomes, therefore it is unlikely to be of benefit to focus scarce resources towards dramatically reducing absolute time to delivery. Rather, our data support the idea that clear, timely, and well-supported clinical decision-making may have more influence on perinatal outcomes. Normal working hours also correspond to the highest availability of non-medical services such as technicians, porters and laboratory clinicians. This may therefore also be an independent aspect for the hospital to consider when developing service design to improve perinatal outcomes. Whilst continuously performing emergency cesarean section has significant demands on resource utilisation and may be a non-modifiable limiting factor, it is rational to believe that clinical delays to treatment are modifiable even within resource constraints. A previous study, for example, demonstrated that the average time to complete obstetric triage reduced from 192 to 38 min when a midwife was allocated to this specific task [22].

The high volume of deliveries and baseline incidence of adverse events at Mulago Hospital meant that, although temporally short, our study was sufficiently powered for the crucial outcomes of perinatal and neonatal mortality. The high baseline incidence of adverse outcomes in our study (~ 5% maternal adverse outcomes and ~ 10% perinatal mortality) are in keeping with previously reported outcomes from the study centre [25, 26]. Furthermore, since such a high volume of deliveries can complicate detailed medical record-keeping, our strategy of bespoke contemporaneous data collection by a dedicated researcher present in the institution increases our confidence in the accuracy of the timings presented. The study is also underpinned by a powerful and sophisticated statistical modelling strategy, in which non-parametric dynamic additive models were used to determine the risks of adverse perinatal outcomes relative to baseline risk, without making assumptions about the risk / time relationship.

A limitation of the current study is that our results relate only to delay in delivery after the decision for emergency cesarean is made. Previous analysis from the study setting has found that the average triage time is longer for mothers who present overnight [22]. Delays besides the decision-to-delivery interval may therefore vary in a predictable diurnal cycle. We were also unable to explicitly model the influence of the experience of available obstetricians throughout the day. Whilst the periods during which the least experienced obstetricians are alone in the hospital correlate with the longest decision-to-delivery intervals and highest risk of perinatal mortality, we could not demonstrate a causal association here. A further limitation is the absence of a routinely applied categorisation of urgency for non-elective caesareans in the study context. Adoption of such a system could help to identify and prioritise higher risk cases, providing further scope for reducing perinatal mortality. While our study utilises a large cohort, there was insufficient power to analysis the relationship between decision-to-delivery interval and perinatal outcome separately for each indication for caesarean section. It might be expected that for some indications, particularly those in which delivery is very urgent such as cord prolapse, that a more direct relationship might exist. For other indications, such as fetal malpresentation, the findings of sub-group analyses would be likely consistent with the full cohort data.

In this context, where many mothers present to hospital already in obstructed labour [27], a direction of future study would be to investigate delays in the total time to delivery besides the decision-to-delivery interval. Such delays may also vary diurnally, for example, due to traffic patterns in the surrounding urban area. Clinician experience can be associated with perinatal outcomes both indirectly, through increased decision-to-delivery interval and directly, through for example operative skill. Further analysis of the obstetricians available throughout the day is required to determine whether modifying the periods during which the those least experienced are alone in the hospital would result in reduced risk of perinatal mortality. Future analyses in this area should also include analysis of factors potentially contributing to adverse outcomes after the decision for emergency caesarean section has been made.

## Conclusion

In this busy sub-Saharan Africa maternity setting, the average decision-to-delivery interval of emergency cesarean section is longer than target times adopted in well-resourced obstetric settings. There is no direct relationship between the interval and adverse perinatal outcomes, however, there is significant diurnal variation in the risk of perinatal and neonatal mortality. The rate of adverse perinatal outcomes is better correlated with time of decision-making than with length of decision-to-delivery interval or time of delivery. This suggests that focus on supporting safe clinical decision-making during high-risk periods may be a useful and feasible strategy for reducing neonatal morbidity and mortality.

## Data Availability

The datasets generated and analysed during the current study are not publicly available due to the terms of the ethical approval under which they were obtained, but may be available from the corresponding author on reasonable request.

## Abbreviations

HR: Hazard ratio
OR: Odds ratio
CI: Confidence interval
